# First Report of *Mycobacterium bovis* and *Nocardia* spp. Co-Infection in a Roan Antelope

**DOI:** 10.3390/ani16111721

**Published:** 2026-06-04

**Authors:** Luca Botta, Matteo Cuccato, Neva Cormio, Veronica Crocchianti, Maria Goria, Emanuelle Bergeron, Delphine Mouniée, Veronica Rodriguez Nava, Frine Eleonora Scaglione

**Affiliations:** 1 Department of Veterinary Science, Università degli Studi di Torino, Largo Paolo Braccini, 2, 10095 Grugliasco, Italy; matteo.cuccato@unito.it (M.C.); neva.cormio@unito.it (N.C.); frineeleonora.scaglione@unito.it (F.E.S.); 2 Veranex Preclinical Services, 42 Bd Jourdan, 75014 Paris, France; veronica.c@veranex.com; 3 Istituto Zooprofilattico Sperimentale del Piemonte, Liguria e Valle d’Aosta, Via Bologna 148, 10154 Turin, Italy; maria.goria@izsplv.it; 4 “Bacterial Opportunistic Pathogens and Environment” (BPOE) Research Group, CNRS 5557, INRAE 1418, VetAgro Sup, Université Claude Bernard Lyon 1, UMR Ecologie Microbienne—Lyon (LEM), 69280 Marcy l’Etoile, France; emmanuelle.bergeron@univ-lyon1.fr (E.B.); delphine.mouniee@univ-lyon1.fr (D.M.); veronica.rodriguez-nava@univ-lyon1.fr (V.R.N.)

**Keywords:** roan antelope, granulomatous pneumonia, *Mycobacterium bovis*, *Nocardia tengchongensis*

## Abstract

*Nocardia* spp. and *Mycobacterium* spp. are known etiological agents of granulomatous pulmonary infections in humans and animals; however, co-infections involving these pathogens have not previously been reported in veterinary medicine. This paper describes the first documented case of co-infection with *Mycobacterium bovis* and *Nocardia* spp. in a captive roan antelope (*Hippotragus equinus*). The animal was a 9-year-old female roan antelope from a safari park in northern Italy that died suddenly with a one-month history of weight loss. Post-mortem examination revealed severe, diffuse, chronic granulomatous pneumonia associated with fibrino-granulomatous pleuritis and granulomatous pericarditis. Histologically, multifocal to coalescing necrotizing granulomas were observed, with intralesional acid-fast bacteria. Microbiological culture and biomolecular analyses allowed the identification of *M. bovis* and *Nocardia* spp. in lung tissue samples. The *Nocardia* genome sequence was 98.5% similar to *N. tengchongensis*, a recently discovered species. The findings emphasize the importance of comprehensive diagnostic approaches in animal granulomatous lung disease. Mixed infections in captive wildlife represent a One Health concern, as the potential for zoonotic adaptation and transmission to humans cannot be excluded. Therefore, pathogen surveillance is of particular importance within zoological collections.

## Simple Summary

This study reports an unusual pulmonary infection in a captive roan antelope, a species commonly housed in zoological collections. The animal showed progressive weight loss for about one month and died suddenly. Post-mortem examination revealed multifocal whitish pulmonary and pleural nodules. Microscopic examination showed severe diffuse granulomatous pneumonia. Laboratory analyses identified *Mycobacterium bovis* and *Nocardia* spp. Sequencing showed 98.5% similarity with *N. tengchongensis*, a recently discovered environmental bacterium never previously associated with animal disease. This represents the first documented co-infection with these two bacteria in a roan antelope. The influence of environmental exposure and weakened immune defenses could not be ruled out. This case highlights the importance of careful health monitoring in zoo animals and raises awareness of potential risks at the interface between wildlife, the environment, and human health.

## 1. Introduction

*Nocardia* spp. and *Mycobacterium* spp. are widely recognized causative agents of pulmonary infections in both humans [1] and animals [2]. In both hosts, pulmonary involvement usually corresponds to granulomatous pneumonia [3]. Although immunocompromised individuals are more prone to developing severe disease, infections caused by these pathogens have also been occasionally reported in immunocompetent hosts [3].

Among *Mycobacterium* species, *M. bovis* is a well-known etiological agent of granulomatous pneumonia in animals and it is considered the primary agent of cattle tuberculosis [4,5]. The target organs are usually the alimentary and the respiratory tract, as the bacteria enter the host mostly via inhalation or ingestion and are subsequently disseminated throughout the whole body [6].

Similarly, the genus *Nocardia* has also been previously associated with granulomatous disease [7]. These bacteria are ubiquitous environmental organisms, and infection typically occurs via traumatic tissue penetration or, more commonly, by inhalation of contaminated particles [8]. New species of the genus have recently been discovered, such as *N. tengchongensis*; however, to the authors’ knowledge, no clinical case in humans or animals has been associated with this *Nocardia* species.

Co-infections with *Nocardia* spp. and *Mycobacterium* spp. have been previously reported in human medicine, with a wide variety of affected organs, specifically endocrine glands [9], central nervous system [10], and lungs [11,12]. Both *M. tuberculosis* and *M. non-tuberculosis* complex were observed in pathological specimens, while several *Nocardia* species have been reported, without the predominance of a single species. To the authors’ knowledge, no cases of *Mycobacterium* spp. and *Nocardia* spp. co-infection have been documented in veterinary medicine.

Pulmonary manifestations of granulomatous infections have also been reported in both captive and wild antelopes, where affected animals presented with conglomerate lobar pneumonia and mediastinal lymph nodes tubercules [13,14]. In these cases, the etiological agent was identified as *M. bovis*.

The roan antelope (*Hippotragus equinus*) belongs to the group of horse-like antelopes and, due to its adaptation to mesic savannah habitats, is commonly kept in zoological collections, where the influence of anthropogenic pressure is relatively limited [15]. However, zoos represent important animal–human interfaces, where pathogen transmission between species has been documented [16,17]. In particular, Lee et al. reported an outbreak of *M. bovis* in a Korean zoo in several groups of people [18]. In zoological collections, transmission to humans most commonly occurs through inhalation of infected aerosol particles during close animal management activities, although infection through wounds or skin lesions has also been reported, particularly during animal examinations or treatments [18]. Therefore, captive facilities represent significant One Health concerns, as they may facilitate pathogen maintenance, cross-species transmission, and potential zoonotic adaptation. This paper describes a case of co-infection with *M. bovis* and *Nocardia* spp. in a captive roan antelope. The species of *Nocardia* showed high similarity with *N. tengchongensis*, a recently discovered species of the genus that has not been associated with clinical cases yet.

## 2. Materials and Methods

In August 2023, a 9-year-old female roan antelope was submitted for necropsy at the Department of Veterinary Sciences, University of Turin, after the signing of an informed consent. The animal originated from a safari park in northern Italy, where it was transferred in 2016 after being born in Germany.

The antelopes were living freely in a shared habitat, alongside other Perissodactyla and Artiodactyla. A regular prophylaxis against clostridiosis and Bluetongue infection was administered to all the individuals of the same species.

The individual had a one-month history of weight loss. Fecal examination revealed the presence of strongyles, treated with four cycles of ivermectin associated with fenbendazole. However, no other clinical investigations were performed. Despite the treatment, no improvement was observed in the animal’s condition, and it died without showing any further symptoms.

Samples from the lungs and pericardium were fixed in 10% buffered formalin, embedded in paraffin, sectioned and stained with hematoxylin and eosin (HE), Grocott methenamine silver (GMS), Periodic Acid–Schiff (PAS), and Ziehl–Neelsen (ZN) stains. Sections of liver with fungal infestation, normal liver and small intestine with paratuberculosis were used as positive controls for GMS, PAS and ZN stains, respectively.

Samples of spleen, lungs, bone marrow, liver and kidney were sent to the Istituto Zooprofilattico Sperimentale del Piemonte, Liguria e Valle d’Aosta (IZSPLV, Turin, Italy), for microbiological analysis, as well as feces for parasitological analysis. Bacterial culture through non-selective conditions and subsequent confirmation tests were performed according to the IZSPLV laboratory routine methods. Bacterial culture was performed on blood agar and Gassner agar, incubated at 37 °C overnight. Following the identification of colonies morphologically compatible with *Nocardia* spp., the genus was confirmed by MALDI-TOF mass spectrometry, additional subcultures on Sabouraud agar and 5% blood agar incubated in a CO_2_-enriched atmosphere, and ZN and GRAM staining.

Following histological observation, samples of formalin-fixed paraffin-embedded (FFPE) pulmonary tissues were sent to the IZSPLV for *Mycobacterium* spp. detection. *Mycobacterium* spp. DNA was extracted from lung tissue using QIAamp DNA Minikit (Qiagen, Hilden, Germany), with *M. tuberculosis* strain H37Rv and *M. bovis* BCG P3 as positive controls.

For *Mycobacterium* species identification, heminested PCR was performed. The use of the Spoligotyping Kit (Biomall.in, Mumbai, India) allowed the discrimination between *M. bovis* and other bacteria of the *M. tuberculosis* complex.

FFPE lung tissue was subsequently sent to the Microbial Ecology Laboratory in Lyon (FR). Primers NG1 (5′-ACCGACCACAAGGGGG-3′) and NG2 (5′-GGTTGTAAACCTCTTTCGA-3′) were used to amplify a 590 bp fragment specific to the genus *Nocardia*, targeting the 16S rRNA gene. PCR amplification was performed using Ready-To-Go PCR beads (Amersham Biosciences, Orsay, France). Briefly, 10 µL of DNA extract, diluted 1:10 in order to reduce the impact of PCR inhibitors, was added together with primers NG1 and NG2 at a final concentration of 1 µM each, in a total reaction volume of 25 µL. All reactions were performed in triplicate. The PCR reaction was performed under the following conditions: 94 °C for 11 min, 40 cycles of 94 °C for 60 s, 55 °C for 20 s, and 72 °C for 60 s, with a final extension step at 72 °C for 10 min [19,20]. *Nocardia farcinica* IFM 10152 was used as a positive control. In addition, a β-globin PCR assay was performed as an internal control to verify the absence of PCR inhibitors in the DNA extracts. PCR products were analyzed by electrophoresis on a 2% agarose gel. A band of approximately 600 bp, consistent with that observed for the positive control, was detected. The resulting amplicon was subsequently sequenced. The double sense sequencing was performed by Microsynth (Vaulx-en-Velin, France) to identify *Nocardia* species. The resulting sequence was compared with reference sequences in the GenBank database.

## 3. Results

### 3.1. Gross Findings

**Figure 1:**
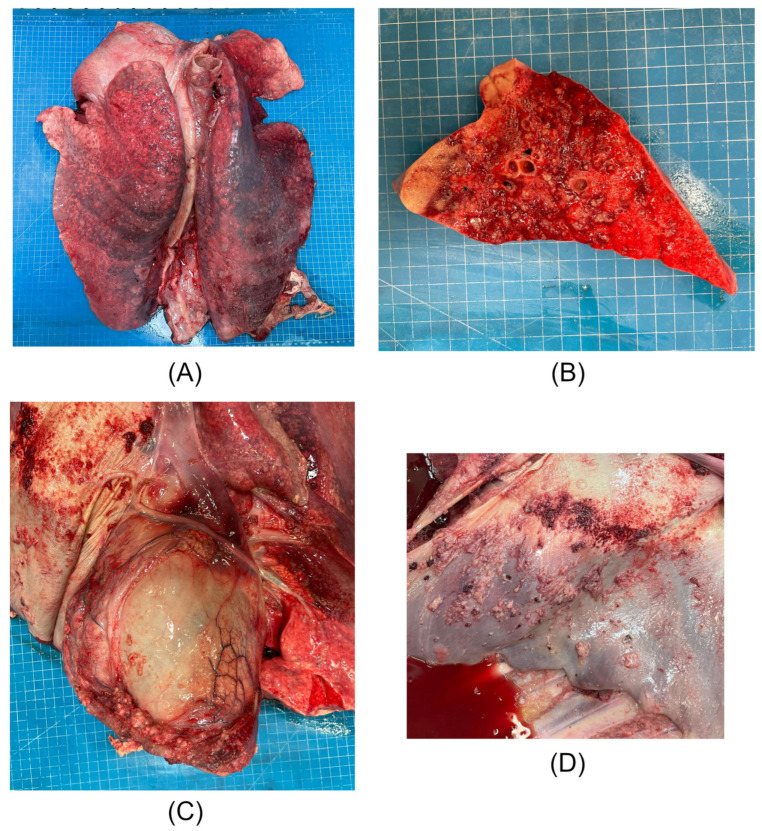
Roan antelope. Gross appearance of granulomatous infection. Background grid squares correspond to 1 cm × 1 cm. (**A**) Numerous small, whitish nodular lesions were diffusely distributed throughout the lung parenchyma; (**B**) Cut section of the lung, presenting the nodules deep in the parenchyma; (**C**) Similar nodular lesions were observed on the entire pericardial surface; (**D**) Diaphragm diffusely covered by abundant fibrinous material, with areas of thickening and adhesions.

At necropsy, a large volume of sero-sanguineous fluid was present within both thoracic and abdominal cavities. The pleural surfaces of both lungs were diffusely covered by abundant fibrinous material, with areas of thickening and adhesions (Figure 1D) and nodular lesions, ranging from 1 to 3 cm. The lungs contained numerous 1–3 cm, whitish nodular lesions diffusely distributed throughout the parenchyma (Figure 1A,B). Similar nodular lesions were observed on the entire pericardial surface (Figure 1C).

### 3.2. Histological Evaluation

**Figure 2:**
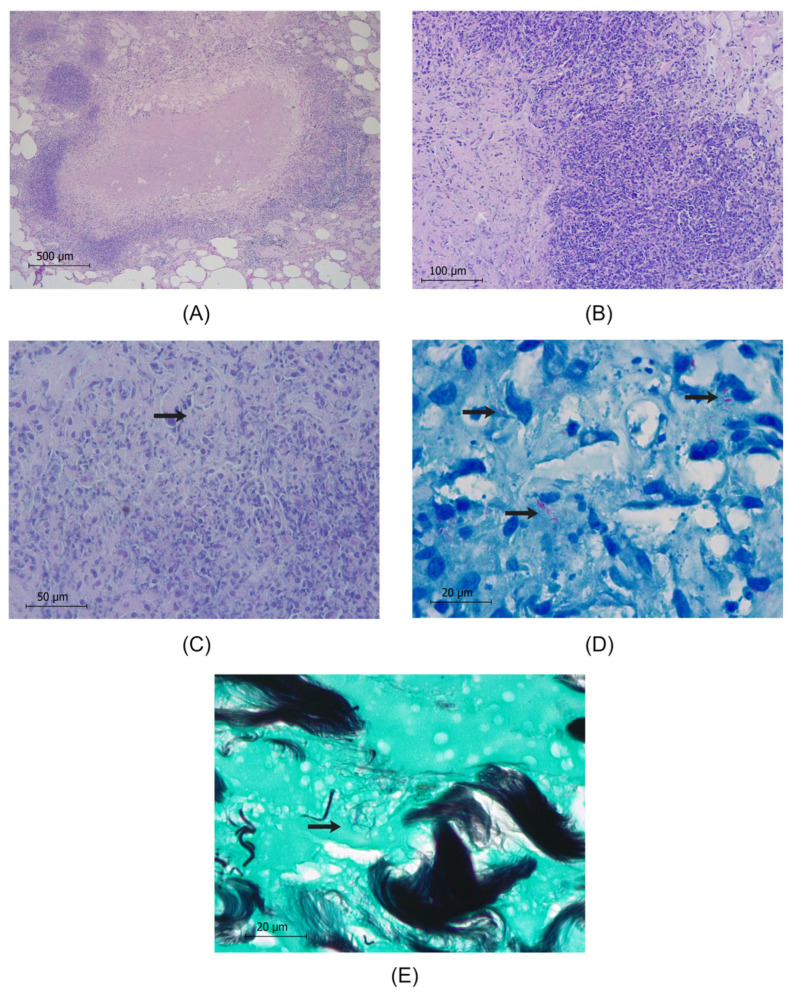
Roan antelope, lungs. Histological appearance of granulomatous pneumonia. (**A**) Pulmonary granuloma, with a necrotic central area composed of eosinophilic cellular debris mixed with fibrine; HE stain, 40×; (**B**) The center of the granuloma is surrounded by a mantle of several macrophages with eosinophilic cytoplasm and paracentral nuclei, as well as several lymphocytes and plasma cells and occasional neutrophils; HE stain, 200×; (**C**) Occasional Langhans-type multinucleated giant cells, up to 10 peripheric nuclei, were observed in the inflammatory mantle (arrow); HE stain, 400×; (**D**) Bacterial colonies stained positively with ZN stain (arrows); ZN stain, 1000×; (**E**) Filamentous bacteria stained positively with GMS stain (arrow); GMS stain, 1000×.

Histological evaluation of the lungs showed multifocal to coalescing granulomas, with a necrotic central area composed of eosinophilic cellular debris mixed with eosinophilic fibrillar material (fibrin) (Figure 2A). The centers of the granulomas were surrounded by a mantle of macrophages with eosinophilic cytoplasm and paracentral nuclei, as well as lymphocytes, plasma cells and occasional neutrophils (Figure 2B). Occasional macrophages with large eosinophilic cytoplasm and up to 10 peripheric nuclei were observed in the same mantle, corresponding to Langhans-type multinucleated giant cells (Figure 2C).

The pulmonary interstitium was diffusely expanded by edema. Reactive fibroblasts and abundant mature collagen fibers were noted near the inflammatory lesions. Diffuse hyperemia of the pulmonary vessels and emphysema were also reported. An analogous inflammatory infiltrate was present on the pleural surface and in the pericardium, with the pericardial adipose tissue being diffusely replaced and expanded by the granulomatous process.

Scattered within the inflammatory cell population, there were intralesional acid-fast bacteria (up to 1 µm in diameter). Bacterial colonies stained positively with ZN stain (Figure 2D). Filamentous bacteria were observed in the GMS-stained slides (Figure 2E). PAS stain was negative.

The morphological diagnosis was consistent with granulomatous pneumonia and severe, diffuse, chronic pericarditis associated with severe, diffuse, chronic-active fibrino-granulomatous pleuritis.

### 3.3. Microbiological and Parasitological Analysis

Fecal examination tested positive for *Nematodirus* spp. eggs.

Bacterial cultures performed at the IZSPLV resulted in positive results in the lung for *Nocardia* spp. and *M. tuberculosis* complex. Mycobacteria were later identified successfully as *M. bovis*.

The presence of *Nocardia* in lung tissue was confirmed, and the sequence was suggestive of *Nocardia tengchongensis*, with 98.5% similarity (GenBank accession number: PX970822).

## 4. Discussion

*Mycobacterium bovis* is widely recognized as a primary etiological cause of granulomatous infections in animals, especially cattle [4,5]. Similar pathological outcomes have been observed in antelopes affected by *M. bovis* infection [13,14,21]. The most common mode of transmission is aerosol spread between herd members [22], an event that may occur in wildlife as well as in captivity, such as in zoological collections. Although aerosol transmission is the predominant pathway, orofecal, congenital, or maternal transmission routes have also been documented [4]. Furthermore, several other species may act as spillover hosts; the impact of this phenomenon is of greater relevance in captivity, where several different species coexist within limited spaces [13,22]. The gold standard for *M. bovis* diagnosis is bacterial culture on pyruvate-enriched selective media [23,24]. However, several studies have shown that PCR results may expand the possibilities of *Mycobacterium* diagnosis [21,25], especially when FFPE tissues are the target of the analysis, as the laboratory technique can also find non-viable organisms [26].

Granulomatous disease is also a clinical manifestation of *Nocardia* spp. infections [7,8]. The bacteria of this genus are capable of colonizing any kind of substrate (water, soil, decaying vegetation, decaying feces) [7]; therefore, animals can be exposed to them via different routes, mainly traumatic tissue penetration, ingestion or inhalation [8]. The clinical respiratory manifestations usually occur in immunocompromised individuals or in patients with chronic lung diseases; however, cases affecting immunocompetent subjects have also been described [1].

In human medicine, several case reports have documented co-infections involving mycobacteria and *Nocardia* spp. Although the lungs were the most affected organs, with pathogens isolated from sputum [21], broncho-alveolar lavage [11,12,21], or lung tissue [12], both agents were also found in thoracic empyema [22], brain abscesses [10], skin abscesses [23], and the thyroid affected by a granulomatous process [9]. *M. tuberculosis* was the most commonly identified mycobacterial species, although sporadic cases led to the isolation of *M. non-tuberculosis* [22], *M. tuberculosis* complex [10] and *M. abscessus* [23]. In contrast, the *Nocardia* species identified in the cited studies were heterogeneous, with no single species predominating, and none of them reported *N. tengchongensis* as an etiological agent.

*Nocardia tengchongensis* was originally isolated in Yunnan province (southwest of China) from geothermally active volcanic soil, characterized by a high content of minerals and organic matter [27]. This type of environment constitutes a favorable reservoir for telluric bacteria capable of adapting to extreme conditions, notably thanks to increased resistance to environmental stresses. These ecological characteristics are consistent with those described for several species of the genus *Nocardia*, known for their persistence in mineral-rich and disturbed soils [1].

In this case, the antelope’s habitat may have played a key role in exposure to *Nocardia* spp. Since the sequenced genome of the observed species presented similarities with *N. tengchongensis*, it is reasonable to consider the zoological environment the most plausible source of contamination, given its similar characteristics. In particular, volcanic substrates or mineral-rich components could constitute a relevant part of the habitat soil, by analogy with the initial isolation medium of the bacterium [27].

The clinical and pathological manifestations observed in this case are consistent with the classical spectrum of nocardiosis [1] and mycobacteriosis, characterized by severe chronic granulomatous pneumonia, which is comparable to the infections described in other animal hosts [28] and in humans [2,7]. Due to the bacterial resemblance to *N. tengchongensis*, this observation could strengthen the hypothesis that *N. tengchongensis* behaves like an opportunistic pathogen, capable of inducing disease when host defenses are compromised or when environmental exposure is sufficient to enable the introduction of the pathogen into the organism. The concurrent *Nematodirus* spp. infestation and the subsequent ivermectin and fenbendazole treatment may have contributed to impaired immune defenses, potentially increasing the antelope’s susceptibility to opportunistic pathogens, such as *Nocardia* spp. [29,30]. The granulomatous lesions associated with *M. bovis* were likely already present as part of a subclinical/chronic process. In this context, *Nocardia* spp. infection may have weakened the animal’s condition, ultimately leading to its death.

In case of granulomatous lung disease, it is important to rule out other potential causes. Several bacterial agents (such as *Rhodococcus*, *Actinobacillus*, and *Actinomyces* spp.) may induce similar lesions [2], and culture remains the gold standard method to reach the definitive diagnosis [5]. Special histological stains (GMS, PAS) are useful to exclude the intervention of fungi (*Blastomyces*, *Cryptococcus*, *Coccidioides*…) [2,3]. It is important to underline that both mycobacteria and *Nocardia* spp. exhibit a positive appearance using the ZN stain. Therefore, next-level diagnostic methodologies, such as genome sequencing, should be implemented in routine diagnostic settings to avoid false negatives [31,32]. Parasitic (i.e., *Dyctiocaulus*), viral, and protozoal agents, as well as noninfectious conditions such as silicosis or foreign body inhalation, should also be considered [2,28].

Virulence factors have been identified in different *Nocardia* species, in particular *N. farcinica* [33,34]. Currently, there are no studies regarding the presence of virulence factors in *N. tengchongensis*, although its opportunistic nature may suggest their existence [34]. Although these virulence genes may remain silent in the environment, their expression could be triggered under favorable conditions within the host. As a matter of fact, the contemporary infection by *Nematodirus* spp. may have acted as a predisposing factor, enhancing the combined actions of both bacteria.

Finally, this first description of an animal infection raises questions regarding the zoonotic potential of *Nocardia* infections in zoological environments. Alongside *M. bovis*, for which the transmission from animals to humans and vice versa is confirmed [35], different species of *Nocardia* have been identified as zoonotic [1]. Given the ecological proximity between animals, the environment and humans, it could not be ruled out that this species may also present zoonotic potential, particularly in immunocompromised individuals.

This study presents some limitations. First, a single case of an affected antelope was observed, which restricted the ability to draw broader epidemiological conclusions, as well as the absence of *M. bovis* testing in the other animals living in the same habitat and the lack of more ante-mortem diagnostic procedures. In addition, no environmental sampling was performed, preventing the assessment of *Nocardia* spp. presence in the soil, which could have represented the source of infection. Moreover, the diagnosis of *M. bovis* was not performed using pyruvate-enriched selective media, which is considered the gold standard for its identification. Finally, only a 98.5% sequence similarity was retrieved between the observed *Nocardia* species and *N. tengchongensis*. These limitations highlight the need for further studies incorporating environmental investigation and advanced molecular tools to better elucidate the epidemiology of mixed infections.

This report emphasizes the importance of controlling diseases in zoological settings, where visitors and zookeepers have potentially close contact with infected animals [16]. As shown by this case, even animals with non-specific clinical signs or no symptoms at all may carry zoonotic pathogens; therefore, biosecurity measures are fundamental to avoid unexpected animal-to-human transmission [36].

## 5. Conclusions

To the authors’ knowledge, this paper represents the first report of *Mycobacterium bovis* and *Nocardia* spp. co-infection in a roan antelope. The post-mortem examination revealed severe, diffuse, chronic granulomatous pneumonia, with local extension to the pericardium, consistent with pathological findings previously reported in the literature [21,28]. The observed *Nocardia* showed 98.5% similarity with *N. tengchongensis*, which has not been reported in any clinical case to the authors’ knowledge. Given the well-known zoonotic potential of both *M. bovis* and the genus *Nocardia*, this identification underlines the necessity of pathogen surveillance in zoological collections, where humans come into close contact with animals that may carry uncommon pathogens. Moreover, as suggested by this case, the environment could also represent a source of infection, as its characteristics are more tailored to the hosted animal rather than the animal keepers who work within the habitats on a daily basis.

## Data Availability

The original contributions presented in this study are included in the article. Further inquiries can be directed to the corresponding author.
